# TPMT and HLA-DQ Allelic Variants in Relation to Drug Response, Safety and Need for Therapy Optimization in Pediatric Inflammatory Bowel Disease

**DOI:** 10.3390/children12101334

**Published:** 2025-10-04

**Authors:** Mirjana Stojšić, Ognjen Ležakov, Sanja Ćeranić, Nikola Stojšić, Marko Rajković, Savina Marković, Milica Kovačević, Nina Brkić

**Affiliations:** 1 Medical Faculty, University of Novi Sad, Hajduk Veljkova 3, 21000 Novi Sad, Serbia; mirjana.stojsic@mf.uns.ac.rs (M.S.); stojsicn@gmail.com (N.S.); 907020d24@mf.uns.ac.rs (S.M.); 2 Institute for Child and Youth Healthcare of Vojvodina, Hajduk Veljkova 10, 21101 Novi Sad, Serbia; sanjaceranic.96@gmail.com (S.Ć.); rajkovicm.bn@gmail.com (M.R.); milicakov997@gmail.com (M.K.); ninaa.brkic@gmail.com (N.B.)

**Keywords:** pharmacogenetics, TPMT, inflammatory bowel disease, azathioprine, biological therapy

## Abstract

**Highlights:**

**What are the main findings?**

- Pharmacogenetic variants shape treatment response to azathioprine and anti-TNF agents in pediatric IBD.
- Genetic differences influence both efficacy and risk of adverse events.
- Evidence remains limited and heterogeneous.

**What is the implication of the main finding?**

- Pharmacogenetics paves the way for personalized IBD therapy in children, but further clinical validation is still required.

**Abstract:**

Background/Objectives: Pharmacogenetics examines genome variability and its influence on drug efficacy and toxicity, forming the foundation for personalized medicine. Patients with inflammatory bowel disease (IBD) treated with azathioprine with thiopurine S-methyltransferase (TPMT) deficiency are at an increased risk of drug-related toxic effects. Variability in the HLA-DQA1 and DQB1 alleles may lead to an inadequate therapeutic response. This study aimed to determine the significance of TPMT and HLA-DQ Allelic Variants in therapy optimization planning. Methods: A retrospective study was conducted to determine TPMT gene polymorphism and the presence of HLA-DQA1 and HLA-DQB1 alleles in children diagnosed with IBD and treated at the Institute for Child and Youth Health Care of Vojvodina in May 2023. Results: The study included 104 children with a mean age of 13.71 ± 3.1 years, with a balanced gender distribution. A TPMT mutation was identified in only one child. The most common HLA-DQA1 alleles were *01 (49%) and *05 (28.8%), while the most frequent allele at the HLA-DQB1 locus was 03 (15.4%). The presence of the HLA-DQA105 allele was associated with the development of anti-drug antibodies against anti-TNF therapy (RR: 1.23; 95% CI: 1.03–1.50), while the presence of HLA-DQA101 was significantly more frequent in children on optimized therapeutic regimens (RR: 1.63; 95% CI: 1.13–2.10). Conclusions: Prior to the initiation of azathioprine therapy, TPMT genotyping should be performed to prevent adverse effects and ensure optimal drug dosing. Identification of the HLA-DQA105 and HLA-DQA101 alleles plays an important role in the planning of biological therapy regimens, including decisions on dose escalation or interval shortening.

## 1. Introduction

Pharmacogenetics bridges the human genome and the proteins that determine drug pharmacokinetics and pharmacodynamics, offering the potential for individualized treatment and the advancement of personalized medicine [1]. Inflammatory bowel disease (IBD)—encompassing Crohn’s disease (CD) and ulcerative colitis (UC)—is a group of chronic autoimmune disorders affecting the gastrointestinal tract (GIT). These conditions result from an inappropriate mucosal immune response to intestinal microbiota in genetically predisposed individuals [2]. Characterized by alternating periods of remission and disease activity, IBD that begins in childhood requires an effective therapeutic strategy for long-term disease control [3].

Thiopurine drugs (e.g., azathioprine) and methotrexate have been used for over three decades as immunomodulators and remain key options for maintaining remission [4]. Azathioprine monotherapy is used to maintain remission in corticosteroid-dependent patients, treat penetrating or perianal disease, and prevent postoperative recurrence.

The introduction of anti-tumor necrosis factor (anti-TNF) agents at the end of the 20th century revolutionized the treatment of autoinflammatory conditions. These drugs are commonly administered subcutaneously (adalimumab, golimumab) or intravenously (infliximab). As the most advanced form of treatment for autoinflammatory diseases, anti-TNF agents allow for mucosal healing, sustained disease control, and have been uniquely effective in closing perianal fistulas in CD patients. They have also been associated with improved growth in pediatric patients treated with infliximab [5]. Anti-TNF agents are primarily used to induce and maintain clinical remission.

A superior therapeutic strategy involves combining azathioprine with an anti-TNF agent, which enhances treatment efficacy by reducing immunogenicity and the formation of anti-drug antibodies, thereby lowering the risk of secondary treatment failure.

### 1.1. Pharmacogenetics of Azathioprine

Azathioprine acts as a purine antagonist, inhibiting DNA and RNA synthesis and, consequently, protein synthesis. It is used primarily in the treatment of IBD, but also in systemic lupus erythematosus and rheumatoid arthritis. Despite the availability of newer biological therapies, azathioprine remains a cornerstone of IBD management [4]. Notable adverse effects include bone marrow suppression, nausea, vomiting, hepatotoxicity, and pancreatitis. Azathioprine is a prodrug, converted in vivo into its active form, 6-mercaptopurine. It is inactivated in the liver via xanthine oxidase, while in extrahepatic tissues, its catabolism depends on thiopurine S-methyltransferase (TPMT) and inosine triphosphate pyrophosphatase. Due to this complex metabolism, azathioprine is virtually undetectable after oral administration [6].

Patients with mutations in the TPMT gene may exhibit enzyme deficiency, putting them at risk of toxic metabolite accumulation. Based on genotypes, individuals are classified as normal, intermediate, or poor metabolizers. Normal metabolizers have two functional alleles; intermediate metabolizers are heterozygous with one functional and one nonfunctional allele; poor metabolizers have two nonfunctional alleles. The latter group is at risk of severe bone marrow suppression and potentially life-threatening complications [7]. Approximately 0.3% of the general population carries homozygous TPMT mutations, requiring discontinuation or avoidance of azathioprine. Heterozygous individuals require dose reduction. TPMT deficiency is the leading cause of thiopurine intolerance in the European population [6].

### 1.2. Pharmacogenetics of Infliximab and Adalimumab

Tumor necrosis factor (TNF) is a cytokine that plays a key role in cell signaling and pro-inflammatory responses. Numerous studies have highlighted the importance of genetic variability in therapeutic outcomes [5]. Polymorphisms in the HLA-DQA1 and DQB1 loci may influence treatment response and predispose patients to adverse events and other autoimmune conditions. If an inadequate response to anti-TNF therapy is observed, optimization may be necessary—either by shortening dosing intervals or increasing the dose. Optimization is typically guided by measuring serum drug levels and anti-drug antibodies, as subtherapeutic dosing may increase immunogenicity [8]. The HLA-DQA1*05 allele, present in approximately 40% of Europeans, increases the risk of developing anti-drug antibodies, often necessitating a switch within the anti-TNF class. The HLA-DQA1*02 allele has been associated with an increased risk of pancreatitis, while HLA-DQB1*02 is more frequently seen in CD patients [9]. Comparative mechanisms, efficacy, safety, and pharmacogenetic considerations of azathioprine, anti-TNF therapy, and their combination can be seen in Table 1.

The aim of this study was to explore potential associations between pharmacogenetics markers (TPMT and HLA-DQ Allelic Variants) in therapy optimization planning, including decisions on dose escalation or interval shortening, drug response and safety for pediatric patients with inflammatory bowel disease.

## 2. Materials and Methods

A retrospective study was conducted by reviewing medical records of all children, who were diagnosed with IBD and treated at the Institute for Child and Youth Health Care of Vojvodina, Novi Sad, Serbia in May 2023, in order to determine TPMT gene polymorphism and the presence of HLA-DQA1 and HLA-DQB1 alleles as well as examine their potential association with adjustments of therapeutic regimens. Collected data included demographic (age, sex), type of IBD—UC or CD, therapy modality and laboratory findings (TPMT mutations and HLA DQA1 and B1 alleles).

The Institute for Child and Youth Health Care of Vojvodina is the only pediatric facility in the Autonomous Province of Vojvodina that managed pediatric IBD cases in the region. Parents or legal guardians of all participants provided informed consent for hospital admission and procedures performed during hospitalization. Data were retrospectively collected from medical records. All patients included in this study had a histopathological diagnosis of IBD.

The data were analyzed using descriptive and inferential statistical methods. Data entry and analysis were performed using IBM SPSS Statistics 18.0. The chi-square (χ^2^) test was used to assess differences in categorical variables, while the Mann–Whitney test was applied for comparisons of non-normally distributed continuous variables. Statistical significance was defined as *p* < 0.05, and high statistical significance as *p* < 0.01. The study was conducted in accordance with the Declaration of Helsinki and approved by the Institutional Ethics Committee of the Institute for Child and Youth Health Care of Vojvodina (protocol code 5754, date of approval 26 November 2024).

## 3. Results

**Figure 1:**
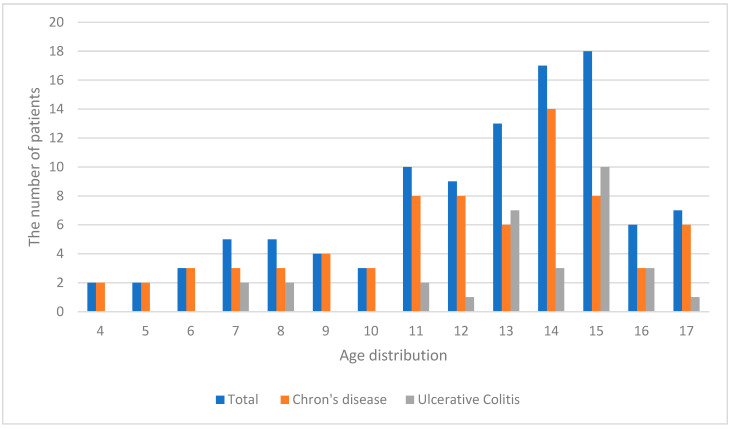
The age distribution of the entire patient cohort, alongside separate distributions for patients with Crohn’s disease and Ulcerative colitis.

This study included 104 pediatric patients whose medical documentation was analyzed. The mean age was 13.71 ± 3.1 years; the youngest patients were 4, and the oldest 17 (Figure 1).

Crohn’s disease was diagnosed in 70.2% and ulcerative colitis in 29.8%, with balanced sex distribution (Table 2). All patients included were receiving biological therapy and were tested for the presence of HLA-DQA1. Due to technical limitations only 41 patients were tested for DQB1 alleles. A total of 44 patients were receiving azathioprine and were tested for TPMT mutation.

Out of 44 tested patients, only one child had a heterozygous TPMT mutation (intermediate metabolizer). No adverse effects were observed during azathioprine therapy, but dosing was adjusted by protocol. Additionally, a post hoc power analysis was performed, showing that, with the existing sample, the power to detect these effects was low (0.15–0.55), below the commonly accepted threshold of 0.80. These results indicate that the study did not have sufficient statistical power to detect small to moderate effects, and therefore the negative findings (no significant association) should be interpreted with caution. For future research, a substantially larger sample size would be required to ensure adequate power.

**Figure 2:**
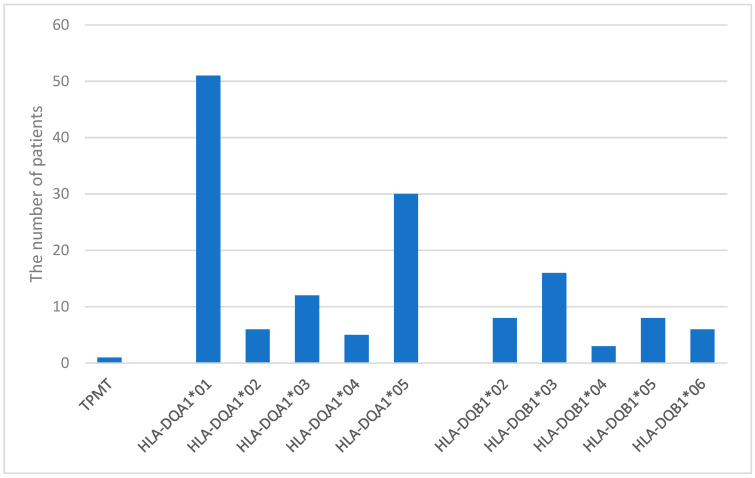
Frequencies of TPMT and HLA-DQA1 and DQB1 alleles in the patient cohort.

The most common HLA-DQA1 alleles were *01 (49%) and *05 (28.8%), while the most frequent HLA-DQB1 allele was *03 (15.4%) (Figure 2).

**Figure 3:**
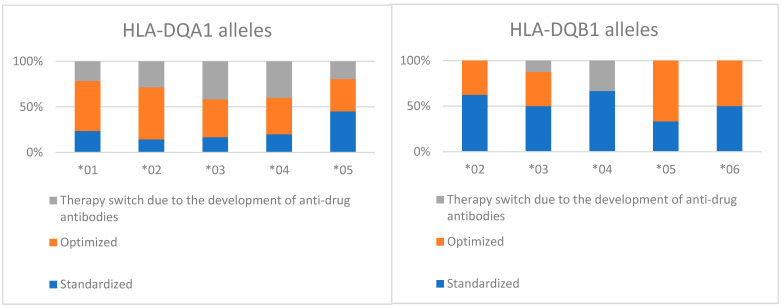
The therapeutic regimens (optimized, standardized and the need for therapy optimization) in relation to the number of patients (percentage) with different HLA alleles. DQA1 alleles shown on the left and DQB1 on the right.

The presence of the HLA-DQA1*05 allele was associated with the development of anti-drug antibodies to anti-TNF therapy (RR: 1.23; 95% CI: 1.03–1.50). HLA-DQA1*01 was significantly more common in patients receiving optimized therapy (RR: 1.63; 95% CI: 1.13–2.10) (Figure 3).

A post hoc analysis was performed, showing the power of ~0.60 for HLA-DQA1*03, indicating that there is still a risk of type II error. For all the other genes, the power is low (0.09–0.27), suggesting that the lack of significant results may be explained by the small sample size and insufficient detection power. The absence of significant differences is therefore likely due to low power rather than a true lack of effect. For future studies, to achieve 80% power, the required size ranges from ~150 to ~1500.

## 4. Discussion

Pharmacogenetics examines genome variability and its impact on drug efficacy and toxicity. The sequencing of the human genome in the 1990s was a milestone in the advancement of personalized medicine [10]. This opened new avenues for individualized treatment strategies, not only in IBD but in other chronic diseases requiring lifelong therapy.

**Figure 4:**
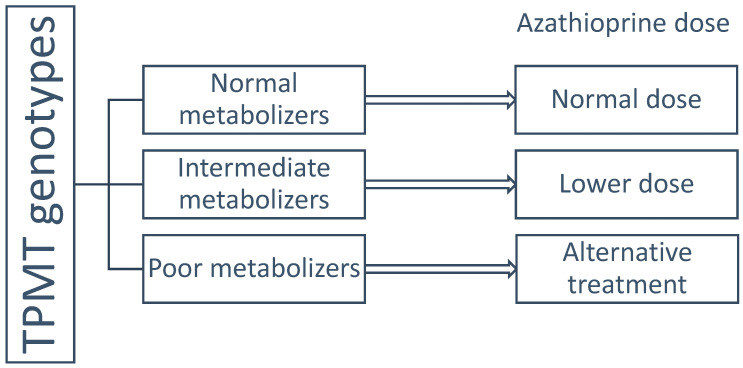
Flowchart representing proposed pharmacogenetic-guided treatment with azathioprine in children with IBD, based on TPMT genotype.

The majority of patients in our cohort were adolescents (children older than 12). This is consistent with the known epidemiology of inflammatory bowel disease, which shows a peak incidence during adolescence [2,3]. In our study population, only one patient had a heterozygous TPMT mutation. According to Baker et al., routine TPMT genotyping should be considered prior to azathioprine initiation to prevent adverse effects and potential underdosing [6]. In a study involving 99 CD patients and 32 with UC, TPMT activity was assessed before starting azathioprine. Among four patients who developed myelosuppression, none had low TPMT activity; one had intermediate, and three had high activity. Nevertheless, routine genotyping was recommended, as enzyme activity can decline after therapy initiation [11]. The FDA (Food and Drug Administration) also recommends TPMT genotyping prior to thiopurine therapy [6], and CPIC (Clinical Pharmacogenetics Implementation Consortium) guidelines advocate for genotype-based dosing adjustments [7]. A retrospective German study of 93 IBD patients reported that 15% discontinued azathioprine due to adverse effects. Most (nine patients) had gastrointestinal toxicities without TPMT mutations. However, one patient with complete TPMT deficiency and two heterozygous carriers developed pancytopenia, supporting the need for TPMT testing to avoid potentially fatal myelotoxicity [12]. The same was confirmed by Tavano et al. analyzing 383 Italian adult IBD patients. CD patients with leukopenia had a higher frequency of the TPMT haplotype (*p* = 0.024). These findings supported the importance of TPMT assessment to mitigate azathioprine-related leukopenia in IBD patients [13]. Therefore, TPMT testing should be considered a mandatory part of clinical practice, as per recommendations of the cited studies (Figure 4).

Anti-TNF therapy has become a mainstay in pediatric IBD management. However, due to its immunogenicity, repeated administration can trigger an immune response and antibody formation. HLA-DQA1*05 significantly increases this risk.

The 2016 PANTS (Personalized Anti-TNF Therapy in Crohn’s disease) protocol, based on a three-year prospective study of over 1000 patients, established a strong link between HLA-DQA1*05 and anti-drug antibody development. This allele identifies patients who are not suitable for anti-TNF monotherapy. For these individuals, adalimumab (lower immunogenicity) is preferred over infliximab, ideally in combination with an immunomodulator [14]. Our study showed similar findings (RR: 1.23; 95% CI). Gonzales et al. conducted a retrospective study of 150 patients, confirming that secondary loss of therapeutic response was associated with the presence of HLA-DQA1*05 [15]. Interestingly, we found that the HLA-DQA1*01 allele was significantly more common in children requiring therapy optimization (dose escalation or interval shortening), although this association has not been described in the literature.

Our observations regarding HLA-DQA1*05 are consistent with research performed by DelBaugh et al. who validated a rapid pharmacogenomic assay for the detection of this allele, enabling timely identification of patients at risk of resistance to anti-TNF therapy [16]. Similarly, the systematic review by Lauro et al. emphasized that the pharmacogenetics of biological agents, particularly HLA variants, may be crucial for predicting therapeutic response and reducing immunogenicity [17]. Nowak et al. demonstrated that the presence of HLA-DQA1*05 is also associated with a more severe disease phenotype, namely extensive ulcerative colitis at diagnosis in children [18]. These findings further highlight the clinical relevance of pharmacogenetic testing in the pediatric IBD population. Additionally, Maksic et al. pointed out that the HLA-DQA1*05 allele plays a significant role in immunogenicity among pediatric IBD patients receiving anti-TNF therapy, particularly infliximab and adalimumab, by increasing the risk of anti-drug antibody formation and loss of therapeutic response. They emphasize that the use of immunomodulators can mitigate this risk [19]. Pau et al. analyzed the involvement of HLADQA1*05 in pediatric patients. They concluded that the HLA-DQA1*05 allele does not increase the risk of secondary loss of response to anti-TNF therapy, which is in contrast to other studies. However, their sample included 65 pediatric IBD patients and the children were receiving combination therapy with immunomodulators, which may have attenuated anti-drug antibody formation [20]. The most common HLA-DQB1 allele in our study was 03, which Boscá-Watts et al. linked to an increased risk of celiac disease, particularly when co-present with HLA-DQA1*03, forming the HLA-DQ8 haplotype. This locus is less common in UC patients [9]. In our sample, it was present in seven patients and evenly distributed across IBD subtypes. Due to the low incidence, a direct link to celiac disease could not be established.

**Figure 5:**
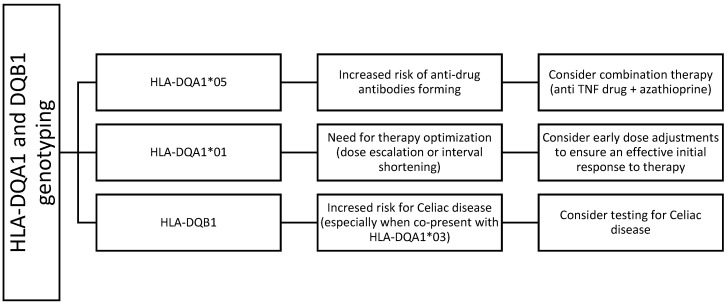
Flowchart representing proposed pharmacogenetic guideline in anti TNF treatment with infliximab and adalimumab, regarding dosing adjustments and combination therapy, as well as potential need for Celiac disease testing, in children with IBD, based on HLA-DQA1 and DQB1 alleles.

Our results, together with evidence from prior studies, support the recommendation for routine HLA testing as an integral component of therapeutic planning in pediatric IBD (Figure 5).

This study has several limitations. The sample size was relatively small, which was proven by the post hoc analysis. Not all patients were analyzed for all genotypes. Although we explored the association between HLA-DQA1*01 and adjustments in therapeutic regimens, other factors may have influenced therapy optimization. Potential confounders such as patient age, sex, disease subtype, disease duration, comorbidities, and concurrent genetic variants could have contributed to treatment decisions. These limitations highlight the need for further research with larger cohorts and multivariate analyses to further assess TPMT mutation significance and to clarify the independent effect of HLA-DQA1*01.

## 5. Conclusions

Pharmacogenetics represents a key step toward the development of personalized therapy. The goal of any long-term treatment is to maximize therapeutic efficacy using the lowest effective dose, thereby improving quality of life, reducing disease burden, and minimizing adverse effects. TPMT genotyping should be performed prior to azathioprine initiation to prevent toxicity and guide appropriate dosing. Given the risk of anti-drug antibody formation, as seen in our research, HLA-DQA1*05 and HLA-DQA1*01 typing provides valuable information for planning biological therapy, including decisions about combining biologics with immunomodulators or adjusting therapy through dose escalation or interval shortening.

## Figures and Tables

**Table 1:** Overview of mechanism, efficacy, safety, and pharmacogenetic aspects of azathioprine, anti-TNF therapy, and their combination.

	Azathioprine	Anti-TNF Agents (Infliximab/Adalimumab)	Combination Therapy (Aza + Anti-TNF)
Mechanism	Purine analog—inhibits lymphocyte proliferation	Monoclonal antibodies against TNF-α	Dual mechanism (immune suppression + cytokine neutralization)
Efficacy	Effective in maintenance, slower onset	High efficacy for induction and maintenance	Improved remission rates compared to monotherapy
Adverse effects	Myelosuppression, hepatotoxicity, pancreatitis	Infusion reactions, infections, immunogenicity	Reduced immunogenicity of anti-TNF, but higher risk of adverse events
Pharmacogenetics	TPMT variants important for dosing	HLA-DQA1*05 linked to anti-drug antibodies	Combination reduces antibody formation

**Table 2:** The distribution of patients with Crohn’s disease and ulcerative colitis and the gender distribution of the patients.

		IBD		
		Crohn’s Disease	Ulcerative Colitis	Total
Gender	Male	39 (37.5%)	14 (13.4%)	53 (50.9%)
	Female	34 (32.7%)	17 (16.4%)	51 (49.1%)
	Total	73 (70.2%)	31 (29.8%)	104 (100%)

## Data Availability

The data presented can be provided on request. The original contributions presented in this study are included in the article. Further inquiries can be directed to the corresponding author.

## Abbreviations

The following abbreviations are used in this manuscript:

CD	Crohn’s disease
CPIC	Clinical Pharmacogenetics Implementation Consortium
DNA	Deoxyribonucleic Acid
FDA	Food and Drug Administration
GIT	Gastrointestinal Tract
HLA	Human Leukocyte Antigen
IBD	Inflammatory Bowel Disease
PANTS	Personalizing Anti-TNF Therapy in Crohn’s Disease Study
RNA	Ribonucleic Acid
TNF	Tumor Necrosis Factor
TPMT	Thiopurine Methyltransferase
UC	Ulcerative Colitis
